# Antimicrobial coatings for orthopaedic implants - Ready for use?

**DOI:** 10.7150/jbji.46508

**Published:** 2020-04-23

**Authors:** Volker Alt, Antonia F. Chen

**Affiliations:** 1 Department of Trauma Surgery, University Hospital Regensburg, Regensburg, Germany; 2 Department of Orthopaedic Surgery at Brigham and Women's Hospital, Harvard Medical School, Boston, USA

Implant-associated infections remain a tremendous challenge, and all efforts should be taken to improve the prevention and treatment of this entity. A critical factor in the establishment of implant-associated infections is the colonization of the implant surface and subsequent biofilm formation by bacteria or fungi in the context of the “race for the surface” 1, 2. The biofilm itself underlies a maturation process. In most cases, mature biofilm formation on an implant surface 3 requires removal of the implant, adequate surgical debridement and antibiotic treatment for cure 4.

Antimicrobial coatings technologies offer the option of local protection of the implant surface against the above-mentioned implant colonization and biofilm formation of microorganisms. Despite tremendous research in this field, there are currently only a few clinically available implants with antimicrobial coatings on the market 5, 6.

## Implant coatings

In the narrow sense, implant coatings include technologies with direct modification of the actual implant surface (i.e.; implemented at existing implant manufacturing lines), which can directly be used by surgeons in the operating room. These technologies mainly consist of systems that release agents with antimicrobial activity such as silver, gentamicin and povidone-iodine. These compounds are attached directly to the implant surface during the manufacturing process by orthopaedic device companies 5. The efficacy of coated implants has been demonstrated in both clinical and animal models 7. The level of evidence, though, may vary between these studies. Similarly, efficacy relates to defined outcome parameters (e.g.; bacterial colony forming units / mL, surgical site infections, periprosthetic joint infections). Larger coated implants, for example, are typically used in orthopaedic oncology 8. Studies deriving from this medical field include data from more than 500 patients treated with silver-coated megaendoprostheses or knee arthrodesis implants. Implants were safe with the only minor risk of development of argyria 5, 9, 10. Randomized controlled trials are still lacking. The available data, however, indicates that silver-coated implants are beneficial in reducing infection rates in high risk patients with megaendoprostheses in revision or tumor bone surgery 9-11. Other coating surfaces include copper, magnesium, and copper-titanium dioxide 12.

## Surface Treatment of Implants

Implant coatings are different from surface treatment of implants by surgeons. In the latter, the surgeons apply a compound directly to the metallic surface of the implant immediately prior to implantation. Hydrogels or other antimicrobial drug delivery systems, such as antibiotic-loaded calcium sulfates and calcium phosphates, belong in this category 6. Most of these agents were not primarily designed for implants, and hence, are not officially approved by authorities for the indication of surface treatment. Two randomized controlled trials on covalently linked hyaluronan and poly-D,L-lactid hydrogel have demonstrated wound healing in fracture cases with significantly less surgical site infections 13, and significantly less surgical site infections in total joint arthroplasty cases with no interference with osteointegration 14. According to today's requirement for randomized controlled trial (RCT) evidence, these data have to interpreted under the light that they were generated by one scientific group only and the trials were not registered in official trial registries (e.g.; *clinicaltrials.gov*).

## Future Coatings

Most experimental coatings involve antibiotics or antimicrobial compounds that have been tested in the laboratory setting. Novel compounds include CZ-01127 that allow elution of high antibiotic concentrations 15 and polymer implant coatings allow for active and immediate antibiotic delivery with additional passive or longer term antibiotic delivery 16. Novel compounds, such as ceragenin-90 (CSA-90) and sphingosine, have been generated to combat antibiotic resistance and prevent and eliminate biofilm from organisms that commonly cause orthopaedic infections 17, 18. These new compounds and release mechanisms have promise to enhance the current antimicrobial methods of antimicrobial coatings.

Regarding direct coating technologies, passive modification of the implant surface design with modification of its morphology or hydrophilic/hydrophobic properties is another theoretical approach to prevent bacterial adhesion and improve the implant's robustness against infection, but none of these technologies have reached market approval yet 7.

## Regulatory requirements

Current regulatory requirements are challenging for the market approval of antimicrobial coated implants in the orthopaedic field 19. On both sides of the Atlantic, coated implants using antimicrobial agents are categorized as Class III Medical Devices and/or Combination Devices since they include as an integral part a medicinal substance (European Medical Device Regulation EU 2017/745, Annex VIII). Under those regulations, manufacturers are required to provide clinical data on safety and performance of a medical device including an overall positive benefit-risk conclusion. Despite an increase in the quantity and quality of published *in vitro* data for orthopedic devices, there is still a lack of human clinical data 20. With the antimicrobial coating an additional risk through potential local and systemic side-effects of the antimicrobial substance is introduced. This requires clinical data to justify that risk. Particularly, the demonstration of clinical performance (efficacy) of an antimicrobial coating, which means a significant reduction of infection rates compared to the standard uncoated implant in a clinical trial, is a considerable barrier due to the required high sample size for a superiority trial design from a statistical point of view.

## Conclusions

Clinical safety of all currently available technologies has been demonstrated. There is growing evidence to reduce infection rates by direct coatings, mainly for certain antibiotics, silver and povidone iodine. However, these data have been mainly derived from case series or cohort studies on megaendoprostheses in orthopaedic oncology and complex revision cases with an elevated (re)infection risk, but not from prospective randomized controlled trials. Alternative “indirect” coatings, (i.e.; implant surface treatment) have not been officially approved as implant coatings. Future technologies, such as covalently bonded antibiotics or silicone-based coatings, are on the horizon but will have to undergo complex regulatory approval processes before their official clinical use.

In conclusion, the given clinical safety of the currently available coatings justifies the use of antimicrobial coated implants in “high risk” cases, but individual risk benefit analysis should be performed for each single patient. The orthopaedic community, industry and regulatory agencies are urged to pave the way for further and sound clinical evaluation regarding clinical efficacy of antimicrobial coated implants in randomized controlled clinical trials.
